# Open e-commerce 1.0, five years of crowdsourced U.S. Amazon purchase histories with user demographics

**DOI:** 10.1038/s41597-024-03329-6

**Published:** 2024-05-13

**Authors:** Alex Berke, Dan Calacci, Robert Mahari, Takahiro Yabe, Kent Larson, Sandy Pentland

**Affiliations:** 1 MIT Media Lab, Cambridge, MA 02139 USA; 2 Princeton University, Princeton, NJ 08544 USA; 3 Harvard Law School, Cambridge, MA 02138 USA; 4 MIT Institute of Data, Systems, and Society (IDSS), Cambridge, MA 02139 USA; 5 New York University Center for Urban Science and Progress, Brooklyn, NY 11201 USA; 6 MIT Connection Science, Cambridge, MA 02139 USA

**Keywords:** Economics, Research data

## Abstract

This is a first-of-its-kind dataset containing detailed purchase histories from 5027 U.S. Amazon.com consumers, spanning 2018 through 2022, with more than 1.8 million purchases. Consumer spending data are customarily collected through government surveys to produce public datasets and statistics, which serve public agencies and researchers. Companies now collect similar data through consumers’ use of digital platforms at rates superseding data collection by public agencies. We published this dataset in an effort towards democratizing access to rich data sources routinely used by companies. The data were crowdsourced through an online survey and shared with participants’ informed consent. Data columns include order date, product code, title, price, quantity, and shipping address state. Each purchase history is linked to survey data with information about participants’ demographics, lifestyle, and health. We validate the dataset by showing expenditure correlates with public Amazon sales data (Pearson r = 0.978, p < 0.001) and conduct analyses of specific product categories, demonstrating expected seasonal trends and strong relationships to other public datasets.

## Background and summary

By making purchases, using mobile phones, and conducting everyday activities, people produce digital traces which are collected by companies. More than a decade of research has revealed how these data can be analyzed to represent human behavior^1–7^, as well as how these data can enhance or enable studies that would otherwise rely on data from surveys, which are typically costly to collect. These include studies on wealth and poverty^8^, socioeconomic status^9,10^, economic opportunity^11^, traffic congestion^12^, and urban planning^5,6^. Researchers have also demonstrated how mobile phone data, as well as geotagged social media posts, can be used to track human migration^13,14^ and map population changes^15^.

Data on consumer transactions from banks and credit card companies in particular have been used to study sociodemographics and mobility^16^ and how these characteristics relate to spending behaviors^17^ and financial well-being^18^. These data sources have also been used to study shopping behaviors^19^ and the predictability of consumer shopping patterns^20^.

Transactions and mobile phone data can also be used to inform times of crisis. This was exemplified through the COVID-19 pandemic. Transactions data were used to study how the pandemic impacted consumption patterns, both in the U.S.^21^ and abroad^22,23^. Many more studies used mobile phone data to quantify the pandemic’s impact on human mobility^24–26^, including a study by a U.S. health agency (CDC) which used mobile phone data to analyze relationships between stay-at-home orders and mobility behaviors that reduced infection spread^27^. Others used mobile phone data to study relationships between mobility and infection rates^28–30^, the economic impacts of mobility restrictions^31,32^, and to improve epidemic models^33–35^. Much of this research was made possible because large platforms with access to location data, such as Apple^36^, Google^37^, Facebook^38^, and location based services (LBS) companies^39,40^, released publicly available mobility datasets to address COVID-19. However, these public releases of COVID-19 mobility datasets represent an exception.

Despite the demonstrated utility of datasets generated by consumers through purchases, these data are generally held privately by the companies that collect them. Researchers using transaction datasets often have privileged access through partnerships with companies.

These datasets will continue increasing in scale as people increasingly use devices and digital services, yet their research potential and use cases remain unrealized. At the same time, traditional data collection through government surveys, which produce statistics and datasets for public use, is in decline. In particular, response rates to important surveys conducted by the U.S. Census Bureau and related agencies have fallen sharply in recent years^41^. Between 2013 and 2023 the response rate for the Current Population Survey (CPS), the source of U.S. statistics on employment, fell by 19%, and the response rate for the Consumer Expenditure Survey, which produces important data on consumer spending and is used to estimate inflation, fell by 15%^42^. Economists have described how prices data collected through new strategies–web scraping, crowdsourcing, purchasing from data aggregators–can be incorporated into official economic indicators to compensate for declining response rates^41^.

Government agencies also conduct surveys from businesses, where response rates have also fallen. As an example, consider the Annual Retail Trade Survey, conducted by the U.S. Census Bureau. Between 2008 and 2018, response rates fell from 82% percent to 64%^41^. In the Technical Validation section of this paper we demonstrate how purchasing data from an e-commerce giant can provide comparable statistics to the Retail Trade Survey.

In a 2020 paper, the Bureau of Labor Statistics (BLS) described how data collected by corporations could benefit the public by improving estimates of the consumer price index (CPI), which is the statistic used to estimate inflation^43^. The CPI is traditionally estimated through a complex combination of surveys and statistical techniques; the paper actively encouraged companies to share prices data in order to reduce sampling error and more effectively use taxpayer dollars.

To help address the demand and utility of consumer expenditure data, and democratize the benefits of data collected from consumers, we crowdsourced and published a dataset containing purchase histories from more than 5000 U.S. Amazon.com users. The data span 2018 through 2022 and each purchase history is linked to survey data with information on participants’ demographics, platform use, lifestyle, health, and more.

We call this dataset “open e-commerce 1.0” because it is the first of its kind and we hope that publishing the data will catalyze future work in this area. Namely, while this dataset can serve a variety of research purposes, its utility will be enhanced when future researchers further collect datasets to complement the present one.

## Methods

The data collection process and data publication were approved by the MIT Institutional Review Board (protocol #2205000649).

**Fig. 1:**
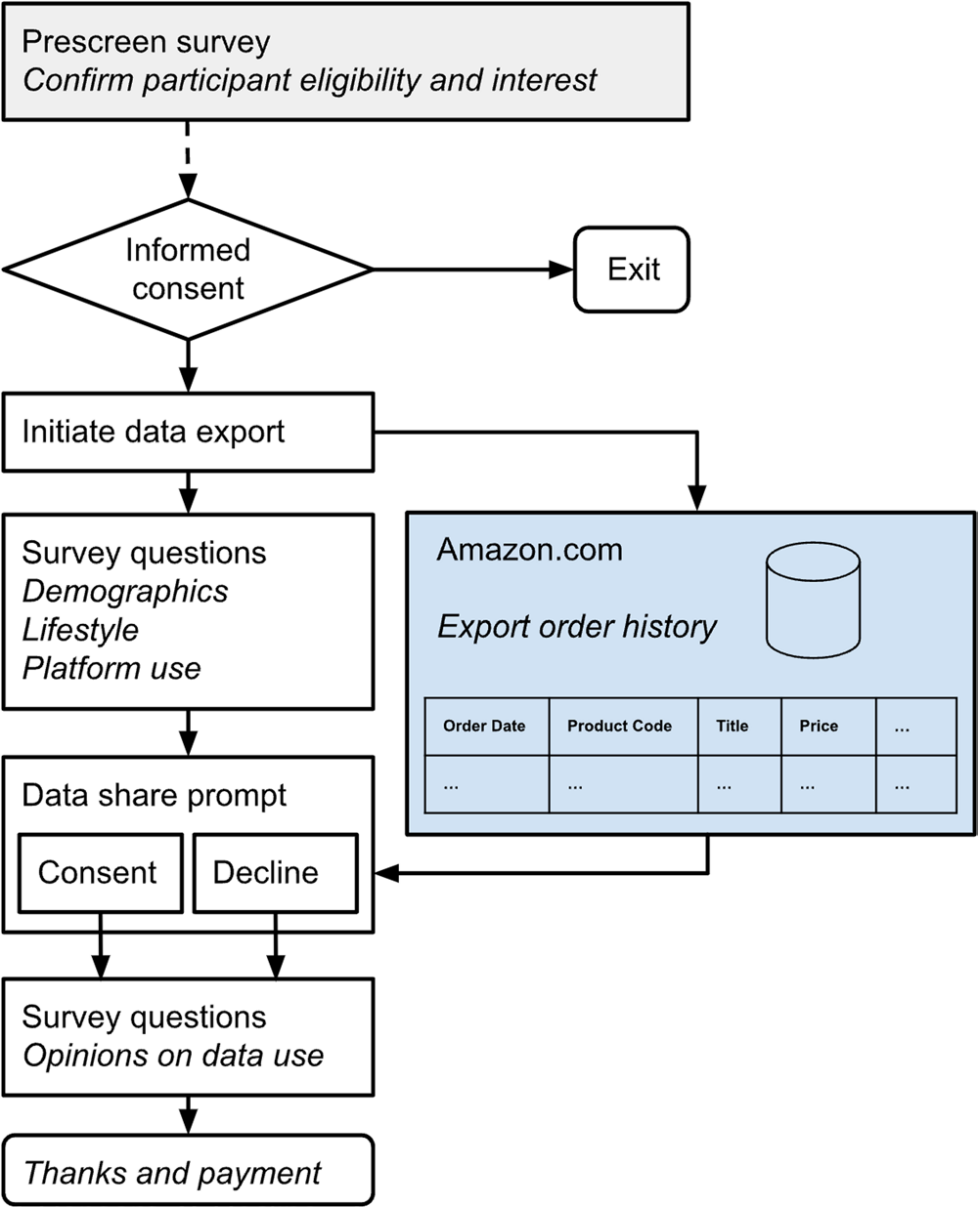
Flowchart representing data collection at a high level.

We crowdsourced the data using a survey tool designed to collect Amazon purchases data from U.S. consumers, as well as participants’ demographics and other user-level variables. The data collection process is summarized at a high level in Fig. 1. Participants shared their Amazon purchases by first exporting their order histories via an “order history reports” page provided by Amazon, which has since been taken offline. (The page was accessible at https://www.amazon.com/gp/b2b/reports). Our survey was designed to prioritize participant consent by allowing participants to opt in to sharing their Amazon data. Care was taken to design a survey tool such that no Amazon data left a participant’s machine without their active consent. Participants were paid whether or not they chose to share their Amazon data.

The survey tool also embedded an experiment designed to test the impact of varying incentives and data transparency levels on share rates, as well as to measure the “privacy paradox”^44^. While noted here, this paper does not cover the experiment–the experiment and results are described in previous work^45^. More information about the experiment design and survey tool can also be found in the Supplementary Information (A).

### Survey design

#### Eligibility requirements

To be eligible for the survey, each participant was required to be a U.S. resident, English speaker, at least 18 years of age, and have an active Amazon.com account that they had been making purchases with since 2018 and that they could log into during the survey.

#### Prescreen survey

A prescreen survey was used to determine whether potential participants met the eligibility requirements. It also contained an attention check and assessed whether participants were interested in the main survey. Participants who passed the attention check and who were determined eligible and interested were invited to participate in the main survey.

#### Main survey

Upon entering the main survey, participants were provided with information about the survey and were asked to affirmatively consent to participation. They were also provided with an outline of the survey which described the Amazon data export process and clarified how participants would have the option to share their data and would be compensated regardless of their choice. It also alerted participants that if they chose to share, their scrubbed data may be made public. Participants were then directed to export their Amazon order history report (purchases data) starting from January 1st, 2018 to the current date they were completing the study (data were collected over the period of November 2022 to March 2023). Since Amazon’s export tool took a variable amount of time to process a request, we designed the survey to enable participants to answer questions while the export request processed.

The survey then asked about demographics, platform use, lifestyle, and health. The survey questions and responses are captured at a high level in Tables 2–6. The precise language used in the survey questions and response options can be viewed through the published survey tool (see the Data Records section). The demographic questions collected information about participants’ gender, age, educational attainment, household income, race and ethnicity, sexual orientation, and U.S. state of residence. Questions then asked participants how many people they shared their Amazon account with, how many people they considered to be in their “household”, and how often they typically ordered deliveries from Amazon. Participants were also asked whether they, or others in their household, experienced any of the following life changes in 2021: moved place of residence, lost a job, became pregnant, had a child, or divorce. Participants were also asked “Are any of the following the case for you or someone in your household or someone you share your Amazon account with?” for questions about marijuana, cigarette, and alcohol use, as well as about having diabetes or using a wheelchair.

**Table 1:** A representative sample of rows from one respondent’s Amazon data.

Order Date	Purchase Price Per Unit	Quantity	Shipping Address State	Title	ASIN/ISBN (Product Code)	Category	Survey ResponseID
2018-01-21	23.07	1.0	OK	OTTERBOX SYMMETRY SERIES Case for iPhone 8 PLUS & iPhone 7 PLUS (ONLY) - Frustration Free Packaging - SALTWATER TAFFY (PIPELINE PINK/BLAZER BLUE)	B01K6PBRSW	CELLULAR_PHONE_CASE	R_2zARigFdY655hAS
2018-02-06	15.91	1.0	OK	Strength in Stillness: The Power of Transcendental Meditation	1501161210	ABIS_BOOK	R_2zARigFdY655hAS
2018-04-03	5.99	1.0	OK	Square Reader for magstripe (with headset jack)	B00HZYK3CO	MEMORY_CARD_READER	R_2zARigFdY655hAS
2018-06-11	4.89	1.0	OK	Dove Advanced Care Antiperspirant Deodorant Stick for Women, Original Clean, for 48 Hour Protection And Soft And Comfortable Underarms, 2.6 oz	B00Q70R41U	BODY_DEODORANT	R_2zARigFdY655hAS

**Table 2:** Sample demographics compared to U.S. census data, with gender, age, household income, education level.

Attribute	Survey (N = 5027)		Census
**Gender**			
Female	2589	51.5%	51%
Male	2311	46.0%	49%
Other or prefer not to say	127	2.5%	
**Age**			
18–24 years	768	15.3%	12.0%
25–34 years	1813	36.1%	17.4%
35–44 years	1240	24.7%	16.8%
45–54 years	677	13.5%	15.5%
55–64 years	374	7.4%	16.1%
65 and older	155	3.1%	22.2%
**Household income**			
Less than $25,000	685	13.6%	17.1%
$25,000−$49,999	1189	23.7%	18.4%
$50,000−$74,999	1063	21.1%	18.6%
$75,000−$99,999	761	15.1%	11.7%
$100,000−$149,999	790	15.7%	14.6%
$150,000 or more	463	9.2%	19.5%
Prefer not to say	76	1.5%	
**Education level**			
Some high school or less	46	0.9%	9.6%
High school diploma or GED	1860	37.0%	55.6%
Bachelor’s degree	2219	44.1%	22.1%
Graduate or professional degree	870	17.3%	12.7%
Prefer not to say	32	0.6%

**Table 3:** Sample ethnicity and race, compared to U.S. census data.

Attribute	Survey (N = 5027)		Census
**Hispanic or Latino**			
Yes	549	10.9%	18.9%
No	4478	89.1%	81.1%
**Race**			
White	3886	77.3%	61.2%
Asian	377	7.5%	5.9%
Black or African American	351	7.0%	12.3%
American Indian and Alaska Native	32	0.6%	1.1%
Native Hawaiian and Other Pacific Islander	5	0.1%	0.2%
Other	105	2.1%	8.6%
Two or more races	271	5.4%	10.6%

**Table 4:** Participant responses to questions about sexual orientation.

	N	%
Heterosexual (straight)	3858	76.7%
LGBTQ+	1111	22.1%
Prefer not to say	58	1.2%

**Table 5:** Survey questions and responses about Amazon account usage and life changes.

Question	N	%
**Number of people share Amazon account with**		
1	3546	70.5%
2	1096	21.8%
3	245	4.9%
4+	140	2.8%
**Household size**		
1	1199	23.9%
2	1590	31.6%
3	983	19.6%
4+	1255	25.0%
**Online purchase frequency**		
Less than 5 times per month	3239	64.4%
5–10 times per month	1407	28.0%
More than 10 times per month	381	7.6%
**Life changes in household in 2021**		
Moved place of residence	1091	21.7%
Lost a job	596	11.9%
Had a child	159	3.2%
Became pregnant	145	2.9%
Divorce	64	1.3%

**Table 6:** Questions and responses about substance use and health.

Question	Answers (N = 5,027)			
*Are any of the following the case for you or someone in your household or someone you share your Amazon account with?*	Yes	No	Prefer not to say	Recently stopped
Smoke cigarettes regularly?	15.0%	81.5%	0.2%	3.2%
Smoke marijuana regularly?	21.1%	75.2%	0.9%	2.7%
Drink alcohol regularly?	44.0%	52.5%	0.5%	3.1%
Have diabetes?	12.3%	87.4%	0.3%	—
Use a wheelchair?	1.9%	97.8%	0.2%	—

Participants then entered the “Data share prompt” section of the survey (see Fig. 1). They were reminded they would be paid whether they consented or declined to share their Amazon data and were prompted to access and potentially share their exported Amazon order history report.

Order history reports from Amazon were exported as CSV files, with a row for each item purchased. Our survey tool collected a specific subset of the CSV columns, which contained no PII. These were: Order Date, Purchase Price Per Unit, Quantity, Shipping Address State, Title, ASIN/ISBN (Product Code), Category. These data fields were explicitly listed for participants. No data from the order history report left participant machines without their consent.

The survey then contained a section of questions asking participants their opinions on how purchase history data should be used. This section also contained an attention check, randomly placed among these questions, so that any participant with a failed attention check could be removed from the dataset in order to improve data quality. Finally, participants were thanked for their time and could optionally insert free-text comments.

### Survey software

The prescreen and main study surveys were implemented using Qualtrics, with a custom software integration that we developed. Our software integration handled processing the Amazon data file within participants’ browsers: It validated the CSV file from Amazon included the specified columns and rows of data representing at least two distinct years, it stripped the file to only include the columns explicitly listed for collection, and it ensured the data did not leave participants’ machines without their consent.

### Data collection

Survey participants were recruited via the online research platforms CloudResearch and Prolific.

We offered prescreen participants $0.35 for an estimated 1 minute survey. We offered participants $1.50 for the main survey with an estimated 4–7 minute completion time. Participants were paid whether or not they opted in to share their Amazon data. However, some participants received additional bonus payments of $0.05, $0.20, $0.50, based on the experimental survey design. More details are in the Supplementary Information (A). Data were collected in a series of batches between November 2022 and March 2023. We stopped collecting data on March 20, 2023 when Amazon took the Order History Reports page offline, which the survey tool depended on.

### Preprocessing

The following procedures were used to preprocess the data to provide the clean and publicly available dataset. We excluded data from respondents with incomplete responses or who failed the attention check (less than 1% failed the attention check in the main survey). Since we recruited participants from multiple platforms, it was possible participants who work on both platforms could participate more than once. We identified duplicates using the Amazon purchases data and dropped corresponding responses from both the Amazon purchases and survey data. We stripped survey data of PII, including the participant IDs assigned by the survey recruitment platform which we used to pay the participants. We also removed free text comments from the survey data to comply with IRB guidelines. The “Shipping Address State” column in the Amazon purchases data had inconsistent values corresponding to the same states. We mapped these values to consistent two-letter state identifiers.

## Data Records

We made the dataset available through Harvard’s Dataverse^46^. This includes the Amazon purchases and survey responses from N = 5,027 participants who chose to share their data. It also includes files to aid data users in understanding the survey questions and responses.

The Amazon purchases and survey responses are provided in separate files, where purchases and survey responses can be linked to a single user by the “Survey ResponseID” column. This “Survey ResponseID” was randomly generated and assigned to survey participants at the start of the survey. By linking Amazon users’ survey responses to their purchases, we can then do analyses of purchases corresponding to the user-level variables. This is demonstrated in the Technical Validation section.

### Amazon purchases

**amazon-purchases.csv** contains all of the collected and preprocessed Amazon purchases from the survey participants who chose to share their Amazon data (N = 5,027).

Each row in this file corresponds to an Amazon order and has the following columns:

- Survey ResponseID
- Order date
- Shipping address state
- Purchase price per unit
- Quantity
- ASIN/ISBN (Product Code)
- Title
- Category

Table 1 shows a representative sample of rows from one respondent’s Amazon data. Note there are rows where values for Title, Category, or Shipping Address State are missing. Shipping Address State is often missing when the purchased item is a digital good, such as a digital gift card, or when the order was delivered to an Amazon locker.

### Survey

**survey.csv** contains the survey responses for the (N = 5,027) participants with Amazon purchases data in this dataset. Note this is a subset of the total survey responses (N = 6,325), since not all participants chose to share their Amazon data. The larger set of survey responses are analyzed and described in another work^45^.

**fields.csv** describes the columns in the survey.csv file, where fields correspond to survey questions. See the descriptive statistics in the Technical Validation section for a high level view of survey questions and responses. The published dataset also includes the survey instrument, which data users can access for more information about the survey interface and logic, and the language used in the survey questions and response options.

## Technical Validation

In this section we first present the demographics and other consumer level variables reported by users who shared their Amazon data through our survey. We then present high level statistics for the Amazon data they shared, and provide analyses to demonstrate how this data is validated by other data sources available. When comparing the reported demographics to U.S. census data, these statistics can be used to assess the representativeness of the dataset. We also demonstrate how the demographic variables can be used to create a stratified sample that is more representative of the U.S. population, to then produce more robust analyses when using the Amazon data. In addition, we present statistics on the other survey question responses, which can help inform further uses of the dataset.

### Descriptive statistics for participant survey responses

Tables 2–4 report on sample demographics with comparisons to U.S. census data when available. Given that eligible survey participants were at least 18 years of age, we compare the sample data to census data for the 18 or older population when possible.

The sample has a slight gender bias with more females versus males, when compared to the U.S. population^47^. This is largely because females chose to share their Amazon data more often than males in the data collection process^45^. Our sample demonstrates an important age bias, under-representing older participants and over-representing younger participants^48^. The sample also under-represents higher-income households, while over-representing middle-income households^49^. Similarly, our sample over-represents individuals with a bachelor’s degree or greater level of education and under-represents those with a high school education or less^49^. For race, our survey allowed selection of multiple categories. When comparing to U.S. census data^50^, we aggregate participants to groups reporting one race category alone or multiple races, and find our sample’s distribution is highly correlated with census data (Pearson r = 0.988, p < 0.001). Even so, participants identifying as Black or African American, Other, or two or more races are underrepresented in our sample. Other data users may wish to aggregate or otherwise handle race groups differently.

Our sample’s geographic distribution is highly correlated with the U.S. population by state (Pearson correlation r = 0.977, p < 0.001)^51^, with exceptions like the absence of survey participants from Puerto Rico and an imbalance in representation from California, Texas, and Pennsylvania. To compute this statistic, we used participants’ survey responses reporting their state of residence in 2021. The proportion of the sample residing in each U.S. state/territory, as well as sample bias when compared to U.S. census data estimates, can be found in the Supplementary Information (B).

In addition to providing demographics, Table 5 reports on survey question responses about Amazon account usage, household size, and life changes. Table 6 reports on responses to questions about substance use and health.

### Descriptive statistics and example analyses with Amazon purchases

#### Descriptive statistics

The Amazon dataset includes 1,850,717 total purchases from N = 5,027 users. Table 7 shows the distribution of the number of purchases per user and Table 8 shows the distribution of the total spend per user when summing over all of their purchases. Table 9 shows the distribution of purchase price per unit for items in the dataset.

**Table 7:** Distribution of the number of purchases per user in the dataset.

mean	368.16
std	426.41
min	1.00
25%	92.00
50%	232.00
75%	489.00
max	5,415.00

**Table 8:** Distribution of the total spend per user in the dataset.

mean	$8,342.06
std	$9,148.10
min	$1.84
25%	$2,199.39
50%	$5,521.61
75%	$11,194.19
max	$110,556.91

**Table 9:** Distribution of unit prices for purchased items in the dataset.

mean	$22.66
std	$46.00
min	$0.01
25%	$8.47
50%	$13.99
75%	$23.75
max	$6,398.95

Table 10 shows the top 5 products by their title, when sorting by the number of distinct users making purchases for the corresponding ASIN/ISBN (Product Code), and when excluding gift cards. Table 11 shows data for the top 5 product categories when aggregating purchases by the “Category” column and sorting by the number of distinct users making the purchases. The tables also report on the total number of purchases and total spend for these categories. Users of the data should note that there are purchases with the same ASIN/ISBN (Product Code) where the “Category” or “Title” differs.

**Table 10:** Top 5 products, number of distinct users purchasing the product, total purchases, and total spend, sorted by number of users, excluding gift cards.

Product Title	Distinct users making purchases	Total Purchases	Total spend
Echo Dot (3rd Gen, 2018 release) - Smart speaker with Alexa - Charcoal	377	484	$13,195.60
Amazon Basics 36 Pack AAA High-Performance Alkaline Batteries, 10-Year Shelf Life, Easy to Open Value Pack	366	571	$6,321.26
Fire TV Stick 4 K streaming device with Alexa Voice Remote (includes TV controls) | Dolby Vision	350	461	$20,670.05
Amazon Basics 48 Pack AA High-Performance Alkaline Batteries, 10-Year Shelf Life, Easy to Open Value Pack	305	576	$8,641.42
Amazon Smart Plug, works with Alexa – A Certified for Humans Device	290	353	$8,428.02

**Table 11:** Top 5 product categories, number of distinct users purchasing products in the category, total purchases, and total spend, sorted by number of users.

Item Category	Distinct users making purchasers	Total Purchases	Total spend
ABIS_BOOK	4236	87,619	$1,359,183.61
ELECTRONIC_CABLE	3521	18,268	$222,390.71
CELLULAR_PHONE_CASE	3468	15,370	$229,662.82
SHIRT	3365	27,267	$514,584.54
HEADPHONES	3307	11,394	$546,323.79

We note there are a significant number of gift card (GC) purchases in the dataset (our categorization of GC includes items with titles containing “gift card”, “gift code”, “digital code”, “Amazon reload”). 40,368 of the 1,850,717 total purchases were for GC’s by 3,220 distinct users and a small number of users made many more gift card purchases than the majority (see Table 12). In particular, the top 99th percentile of GC purchasers (N = 33) made more than 167 GC purchases. We provide further detail on GC purchases in the Usage Notes section, to provide insights on how data users may wish to handle them. The following analyses exclude GC purchases.

**Table 12:** Number of gift card purchases per user in the dataset.

count	3,220
mean	13.37
std	42.27
min	1.00
25%	2.00
50%	4.00
75%	11.00
99%	167.43
max	1,122.00

#### Analyses

Our data collection began in November of 2022 and spanned multiple months, where users were asked to consent to share their data starting from January 2018 to the date of data collection. In order to consistently represent user behavior, the following plots and analyses are limited to data spanning from January 2018 to October 2022. When excluding GC purchases, which is the case in the analyses below, this results in a smaller sample of size N = 5,014 distinct users.

**Fig. 2:**
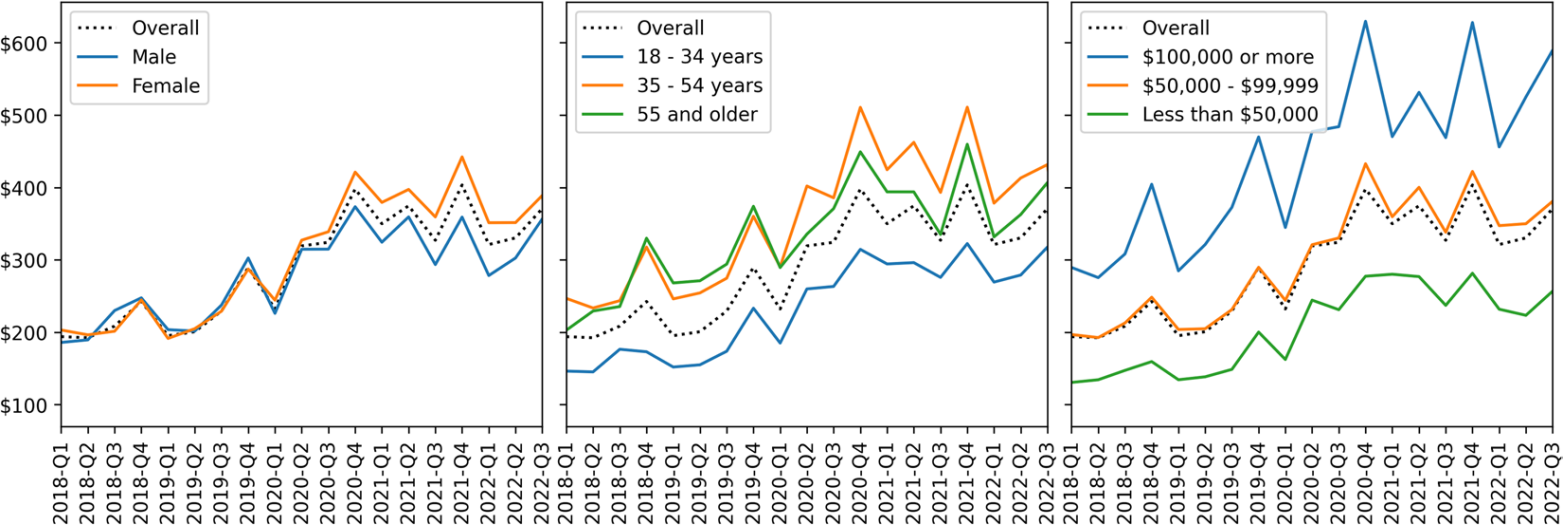
Quarterly median user spend by demographic group, compared to median user spend overall (black dotted line). Left: Spend for Male vs Female users. Middle: Spend by age. Right: Spend by household income.

Figure 2 shows time series plots for the median spend per user, for each quarter, and highlights differences across demographic groups. A gray line shows the median user spend overall. While the demographic groups in Fig. 2 are limited to the Male/Female binary and users who provided their household income, all users, including those who answered “Other” or “Prefer not to say”, are included in the calculation of overall median spend. The left plot shows the difference between Male and Female users. The middle plot shows differences between age groups, where age groups are grouped from the 6 categories collected and shown in Tables 2 to 3 categories: 18–34 years, 35–54 years, 55 and older. The right plot shows differences by household income. Again, categories are grouped from the 6 categories collected and shown in Tables 2 to 3 categories: Less than $50k, $50k - $99k, $100k or more. As might be expected, users with higher incomes spend more on average, especially in the Q4 holiday season. There are also notable differences in spending by age group, where younger users spend less on average, as well as by gender, where female users spend more on average after the start of COVID-19 (2020-Q2). These differences are important given our sample is biased by age and gender, under-representing older adults and over-representing females (Table 2).

With the above differences in purchasing behaviors and sampling biases in mind, we use stratified random sampling, without replacement, to create a stratified sample of users. The strata are defined by a joint distribution of age and sex and match population proportions reported in 2022 U.S. Census data^48^. In particular, strata are defined by a binary definition of sex (Male, Female) and age groups aggregated to 3 levels (18–34, 35–54, 55 and older), as shown in Fig. 2, resulting in 6 strata. The stratified sample has size N = 1,326. The Supplementary Information (C) provides more details on the stratified sampling and displays the sample bias when stratified sampling is not used. The below analyses used to validate the dataset use the larger sample; we use the stratified sample to test the robustness of these analyses.

**Fig. 3:**
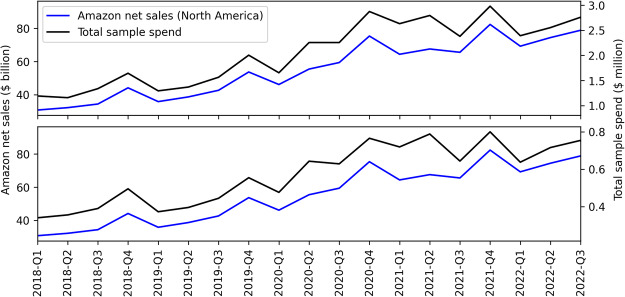
Quarterly Amazon net sales (North America segment) and total user sample spend. Top: Data shown for total spend for the entire sample (N = 5,014). Bottom: Data shown for total spend for the stratified sample (N = 1,326). Data are correlated with Pearson’s r = 0.978 and r = 0.973 (p < 0.001), for top and bottom, respectively.

In order to assess how representative our dataset is for Amazon purchasing in general, we compare Amazon net sales data (for the North America segment) to total spend by users in our sample, for each quarter in our studied period. Figure 3 shows this comparison. The top plot compares Amazon sales data to total spend for our full sample (N = 5,014) while the bottom plot restricts the total spend data to the stratified sample (N = 1,326). Amazon quarterly net sales data are from their quarterly earnings releases produced for investor relations^52^. There are important differences in these sales data sources that we compare: The Amazon net sales data include all of North America, while our purchases dataset is limited to the U.S. Furthermore, our data is for a consistent sample of Amazon users who had accounts in 2018 and does not account for increased sales due to new Amazon users. Despite these differences, the quarterly Amazon sales data and total sample spend are highly correlated. The Pearson coefficient is r = 0.978 (p < 0.001) with data from the entire sample (N = 5,014) and r = 0.975 (p < 0.001) with data from the stratified sample (N = 1,326).

We also assess the representativeness and utility of the Amazon purchases data when considering specific product types. One way we do so is by checking for expected seasonality. GC purchases clearly demonstrate an expected seasonality, with an annual spike in total GC spend in the December holiday season. This is shown in Fig. 7.

**Fig. 4:**
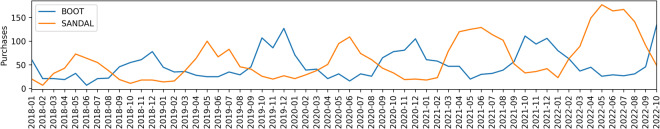
Total purchases each month for categories BOOT and SANDAL. Purchases for these products demonstrate different seasonal trends present in the dataset, where SANDAL purchases peak in summer months while BOOT purchases peak in winter months.

Expected seasonality is also demonstrated in footwear purchases. This is shown in Fig. 4 which plots the total monthly purchases for products in the dataset with category “BOOT” and products with the category “SANDAL”. Total purchases are computed by summing over the quantity in each such purchase row. As to be expected, purchases for these products demonstrate opposite seasonality trends, where SANDAL purchases have yearly peaks in the summer months while BOOT purchases have yearly peaks in the winter months. As a robustness check, we recreate this analysis using the stratified sample and find similar results. This is shown in the Supplementary Fig. D.1.

**Fig. 5:**
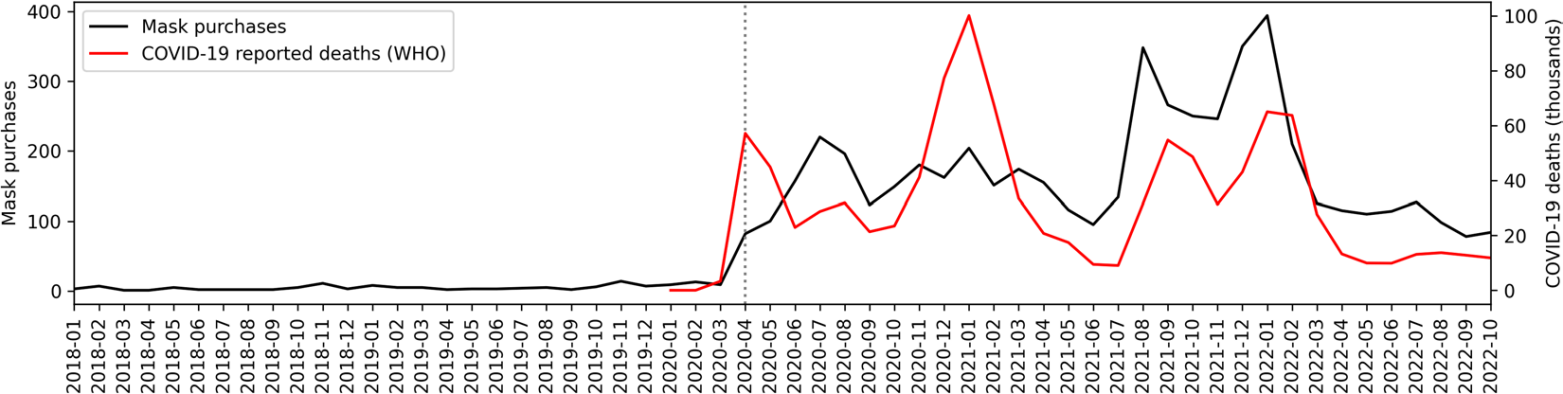
Monthly COVID-19 reported deaths (U.S. data reported by WHO) compared to face mask purchases.

We also validate the Amazon purchases data by demonstrating how purchasing patterns changed in response to the COVID-19 pandemic, using publicly available COVID-19 data. Figure 5 shows a timeseries of the monthly reported COVID-19 deaths in the entire U.S. compared to total number of face mask purchases in our dataset. The COVID-19 data are from the World Health Organization (WHO)^53^. More information about the COVID-19 data and face mask purchases is in the Supplementary Information (F). Figure 5 shows how both the face mask purchases and COVID-19 deaths have a clear initial spike at the start of the COVID-19 pandemic in April 2020. These metrics continue to have similar trends, with spikes in the winter months and when students began returning to school in August and September 2021. More generally, this analysis demonstrates how Amazon purchases data can help analyze changes in online purchasing behaviors over time, or changes in relation to events that impact consumers.

**Fig. 6:**
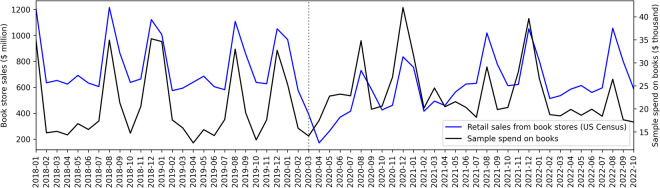
Monthly book store retail sales (from the U.S. Census Bureau) compared to monthly spend on books in the Amazon dataset.

We also compare monthly spend on books in the Amazon dataset to retail sales from book stores. The retail sales data are collected by the U.S. Census Bureau through their monthly retail trade survey^54^. The monthly spend on books in the Amazon dataset is computed over a total of 82,954 book purchases from N = 4180 distinct users. Figure 6 plots time series data making the monthly comparison. It shows how both the retail and Amazon purchases data spike annually in August and December. The plot also displays a change in the relationship of these time series at the start of the COVID-19 pandemic (indicated by a dotted line at month 2020-03).

In order to provide stronger quantitative evidence for the relationship between these time series, we ran an ordinary least squares (OLS) linear regression (Eq. (1)). The regression is constructed to predict the census monthly retail sales data (*retailSales*) from the monthly Amazon user spend data in our sample (*userSpend*). Data are scaled such that retail sales data are in the millions and user spend data are in the thousands of dollars. The regression includes a boolean variable (*postCovid*) taking the value of 0 before the month of 2020-03 and 1 otherwise.

1 $${\rm{retailSales}} \sim {\rm{intercept}}+{\rm{postCovid}}+{\rm{userSpend}}$$

Results are summarized in Table 13.

**Table 13:** OLS linear regression results for Eq. (1) predicting book store retail sales (U.S. Census Bureau data) from Amazon user spend on books (Amazon purchases dataset) for N = 58 monthly observations.

	Coefficient	Std. error	p
Intercept	306.001	60.975	0.000
userSpend	22.367	2.616	0.000
postCovid	−220.075	37.648	0.000

R-squared = 0.630.

As a robustness check, we also perform this analysis with the stratified sample. Results are similar with R-squared = 0.586 and all variables remain statistically significant at the p < 0.001 level). More details about this analysis and the robustness check using the stratified sample are provided in the Supplementary Information (E).

In addition to providing information about product purchases, the Amazon dataset conveys location information: consumers shipped products to their addresses. For privacy reasons, we only collected the shipping address state, and no other address information. From this information, we infer the U.S. state of residence for each user in the dataset for each year of data, and infer when users moved their state or region of residence between years. The U.S. Census Bureau tracks domestic migration between the 4 regions of the U.S. (the Northeast, Midwest, South, West) through the annual American Community Survey (ACS)^55^. This results in 12 data points of population flows between regions for each year. (Each U.S. state is contained within one region.) We compare this census data to migration flows estimated from the Amazon data for the years 2018 to 2019. There is a Spearman correlation coefficient of r = 0.830 (p = 0.001). See the Supplementary Information (G) for analysis details.

## Usage notes

We are publishing this data for research purposes only; the data may not be used to re-identify study participants.

**Fig. 7:**
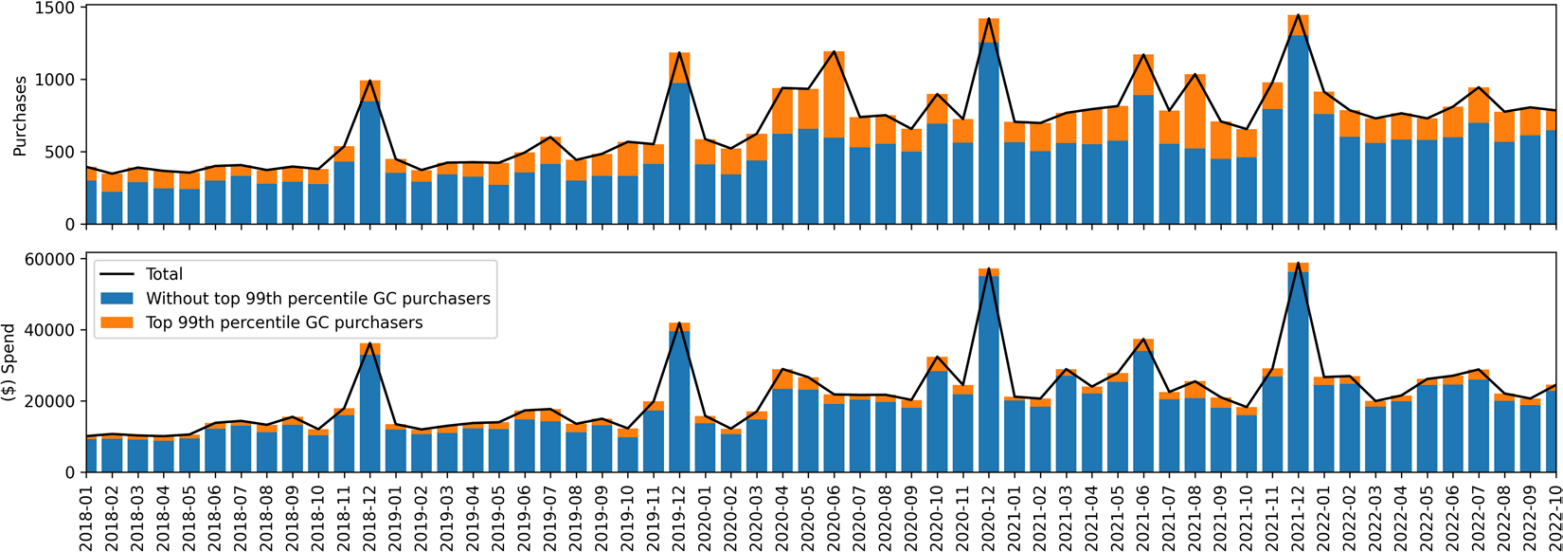
Monthly gift card (GC) purchases. Data for the top 99th percentile of GC purchasers (N = 33) are separated from the other users (orange bars). Top: Total number of GC purchases. Bottom: Total ($) spend on GC purchases.

Before discussing potential future use and limitations, we note users of this dataset should be mindful of a high number of gift card (GC) purchases. The distribution of the number of GC purchases was shown in Table 12. In particular, we identify the top 99th percentile of GC purchasers as those who made more than 167 GC purchases (N = 33). Figure 7 shows how their data disturbs patterns in the number of total GC purchases (top) yet does not greatly impact patterns in total spend (bottom). For example, yearly peaks in expenditure in December are still clear.

A large volume of GC purchases are attributed to very small denominations. For example, $0.50 was among the top 3 most frequently purchased GC denominations in the dataset. (Supplementary Fig. H.1 shows the monthly number of GC purchases for the most frequently purchased denominations.) Researchers using this dataset might take care in handling peculiarities of GC purchases, while other researchers may find them interesting to study.

When using the Amazon purchases dataset, data users may wish to incorporate additional information about the products. While the published data are limited to the columns collected and described in the Data Records section, more information specific to products could be added by collecting information for the product codes (ASIN/ISBN). For example, researchers might be interested in processing the language describing or reviewing products on their associated product pages.

A feature of this dataset is that purchase histories are linked to survey responses that contain consumer demographics and other covariates. These covariates can be used to create reweighted samples that are more representative of the populations researchers wish to study and to perform robustness checks. This was demonstrated in the Technical Validation section where we created a stratified sample based on sex and age. Other reweighting methods or covariates may be better applied for other research applications. For example, survey responses about household size and the number of people sharing the Amazon account may improve analyses sensitive to counting. Beyond strengthening analyses, these covariates might be interesting subjects of study. For example, researchers may be interested in the association between these covariates and product choices or purchasing patterns. One pertinent example of this is prior work that found an association between diabetes (a covariate in our dataset) and the nutritional content in foods purchased from a grocery chain in Greater London^56,57^. Other researchers analyzing the potential risks of current corporate data collection and usage may expose the ease at which protected categories, such as race, or sensitive attributes related to health, may be inferred from purchases data. If these attributes are shown as latent variables within purchases data, such analyses may raise important questions about how these data are transacted in current data markets, or used in black box algorithms.

### Limitations and potential future work

Despite the potential utility of this dataset, as shown in the Technical Validation section, the relatively small size of this dataset will inhibit many compelling analyses and use cases. The dataset represents a small sample compared to datasets available–there are an estimated 163.5 million Amazon Prime users in the U.S. as of Q1 2023^58^ with even more regular online shoppers^59^. We call this dataset “open e-commerce 1.0” because of this project’s aspirations to be joined by more open datasets that will strengthen the utility of the present one.

One example of this limitation is our analysis of domestic migration in the Technical Validation section. While we show a statistically significant correlation between migration estimated from the purchases dataset and census data, the numbers diverge due to our small sample and the fact that few people move between regions each year (<2% of the U.S. population in 2018^55^).

**Fig. 8:**
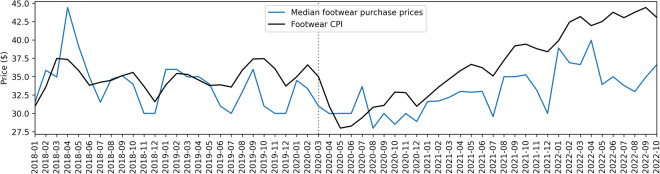
Monthly footwear CPI (U.S. Bureau of Labor Statistics) compared to median prices across footwear products in the purchases dataset. A dotted line marks March 2020 for COVID-19 related changes.

Users of the dataset might also find limitations when addressing use cases described by public agencies that typically have access to larger datasets. For example, a 2020 paper from the Bureau of Labor Statistics (BLS)^43^ describes how the CPI is computed, its important use cases across government agencies (namely estimating inflation), and modernization efforts to incorporate more alternative and corporate data sources into its computation. The paper encourages companies to report price data to the BLS, in order to improve the CPI estimation, to benefit both taxpayers and the business community. The CPI is a complex measure, with price indices computed for a variety of item categories, combined to an aggregate CPI^60^. For some categories, corporate or other alternative data sources are already used. For example, the new vehicles index is estimated using a transactions dataset purchased from the company J.D. Power^61^, and the airline fares index is constructed using fares data from the U.S. Department of Transportation^62^. To illustrate potential use for e-commerce data in computing the CPI, we use footwear prices from the Amazon purchases dataset. Specifically, we compared the monthly footwear price index^63^ to the median price of footwear products in the dataset. These metrics are correlated (Pearson r = 0.536, p < 0.001) with a comparison shown in Fig. 8. Details and further analysis are in the Supplementary Information (I).

Although the metrics exhibit similar monthly patterns, there are important differences. These metrics necessarily differ due to their different data sources. The relatively small size of our dataset is also an important factor. Given more availability of purchases data, more robust price indices could also be derived, especially when tied to consumer demographics, as well as newly innovative price indices. Consumer demographics are important for building metrics representative of populations, but we can imagine more uses of demographic covariates if the wealth of consumer data were available. For example, future research can explore deriving price indices not just specific to item categories, but also specific to consumer categories, such as CPIs by income group, or CPIs specific to different parts of the workforce (e.g. CPIs for service workers, students, retirees) or CPIs more localized to geographic areas or communities. Understanding how price changes and inflation impact these different groups could expand the opportunities for the CPI to serve public agencies and the populace.

An important question going forward is how to expand the present dataset to improve its utility.

Our Methods demonstrated crowdsourcing data from platform users as a means to collecting and democratizing the benefits of corporate data while prioritizing user consent. The data collection tooling we developed is open source (see Code Availability), with the surveys included in the dataset publication. Future researchers are encouraged to collect datasets to complement the present one using these or related strategies. Further research should continue to explore methods to publish platform/corporate data that similarly respect the privacy and informed consent of platform users.

### Supplementary information

Supplementary Information

## Data Availability

All code used to produce the analyses in this paper is available via an open repository: https://github.com/aberke/amazon-study. The repository also includes the survey instruments and custom software used in the data collection process.
