# Supplementary material for: Open e-commerce 1.0, five years of crowdsourced U.S. Amazon purchase histories with user demographics
Source: Sci Data. 2024 May 13;11:491. doi: 10.1038/s41597-024-03329-6 (PMC11091104; doi:10.1038/s41597-024-03329-6)
Supplement: Supplementary file 1 — Supplementary Information [file 41597_2024_3329_MOESM1_ESM.pdf]

Supplementary Information

A. Survey tool and experiment design

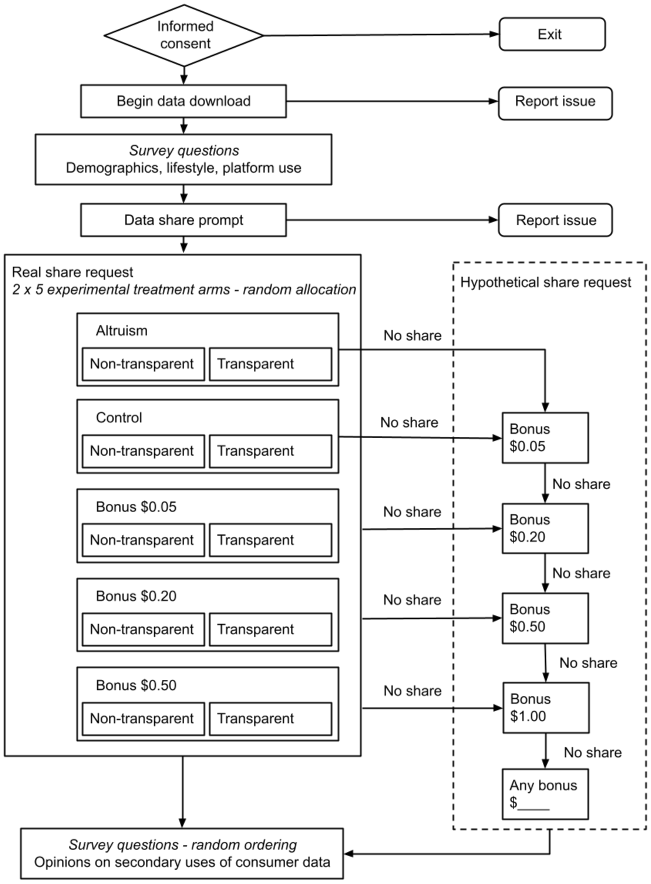

Figure A.1. Flowchart representing the survey. Each box represents a discrete section of the survey. Arrows represent movement from one section to another. “No share” indicates the flow if a participant declined to share within any treatment arm. Boxes within “Real share request” such as “Control” or "Bonus $0.05" correspond to experimental treatment arms that participants were randomly assigned to. “Transparent” and “Non-transparent” boxes represent random assignment into either the transparent condition, where participants were shown their data before choosing to share, or non-transparent, where participants were shown only column names.

Figure A.1 shows a flow chart representing the survey design. While collecting Amazon data, the survey also conducted an experiment designed measure: (1) the effect of framing and monetary incentives on the likelihood that a participant chooses to share their data, (2) the effect of data transparency on share likelihood, and (3) the difference between real share rates and hypothetical share rates. By capturing data about (3) the difference between real versus hypothetical share rates, we provide a unique empirical study of the "privacy paradox" -- the divergence between expressed and actual privacy behaviors[44].

To study the impact of (1) and (2), the experiment had a 2 x 5 factorial design, resulting in 10 separate experimental treatment arms. There were 5 different "incentive" treatments, and 2 different "transparency" conditions. Participants were randomly assigned to an experimental arm with equal probability upon entering the survey. The survey was identical for all participants except on one page of the survey where they made a choice whether or not to share their data ("Real share request" in Figure A.1). Upon reaching this page, participants had already downloaded (but not shared) their Amazon purchase histories data. When prompted to share or decline to share their data, participants were presented with slightly different interfaces based on their experiment arm.

The 5 different "incentive" treatments included a control and 3 different bonus amounts of $0.05, $0.20, $0.50. Participants in the bonus incentive arms were offered these bonuses in addition to the base pay for the survey, if they chose to share their data. Bonuses were limited to $0.50, after consultation with our IRB, to reduce the risk of bonuses having an adverse impact on more vulnerable participant populations. The fifth incentive treatment, referred to as the "altruism" incentive for brevity, added additional text that framed data sharing as an altruistic act: "...We are crowdsourcing data to democratize access to it as a public good and we are asking for your help…"

The 2 different transparency treatments included a "non-transparent" and "transparent" condition. In both treatments, participants were shown the names of the data fields that would be collected if they chose to share. Participants in the "transparent" condition were also presented with all rows and columns of data that would be collected, within a scrollable interface, before they chose to consent or decline to share.

Participants who declined to share were directed to a series of questions designed to measure the hypothetical amount they would have shared their data for, if any.
These questions asked "Would you hypothetically consent to share your data for a bonus payment of $X? (Since you already declined to share your data, your Amazon data will not be collected if you say yes)". The hypothetical bonus amounts ($X) included the real bonus amounts ($0.05, $0.20, $0.50) as well as $1.00.

The amount that participants were first offered in this hypothetical scenario was determined by their original incentive treatment. For example, participants in the $0.20 bonus condition who declined were asked if they would instead hypothetically share for a $0.50 bonus.
If a participant answered "Yes" they exited the hypothetical share request section of the survey. If they answered "No" they were offered the next highest hypothetical bonus amount. If a participant declined the hypothetical after being offered $1.00, they were then asked "How much would you share your data for?" where they could write in a value or indicate "I would not consent to share my data for any amount." The arrows in Figure A.1 labeled "No share" indicate this user flow.

See prior paper[45] for additional information about the survey tool design and experiment results.

B. Sample geographic distribution

Table B.1 shows the number of survey participants from each U.S. state as well as Washing DC and Puerto Rico, in comparison to population estimates from the U.S. Census Bureau[51]. Census data are for the 18+ population in order to provide a better comparison to the survey participants, who were required to be 18+. U.S. state/territory of residence for each person in the sample was determined based on their survey response reporting state of residence in 2021. Figure B.1 plots the sample geographic distribution compared to census population estimates. The left panel compares the proportion of the survey sample and US population living in each state. The right panel shows the sample bias computed as the sample proportion minus census proportion.

Table B.1. Sample geographic distribution by US state/territory compared to population estimates from the US census.

	survey		census
**US state/territory**	N	%	%
Alabama	67	1.3%	1.5%
Alaska	10	0.2%	0.2%
Arizona	95	1.9%	2.2%
Arkansas	46	0.9%	0.9%
California	505	10.0%	11.6%
Colorado	91	1.8%	1.8%
Connecticut	39	0.8%	1.1%
Delaware	13	0.3%	0.3%
District of Columbia	14	0.3%	0.2%
Florida	327	6.5%	6.8%
Georgia	161	3.2%	3.2%
Hawaii	21	0.4%	0.4%
Idaho	18	0.4%	0.6%
Illinois	216	4.3%	3.7%
Indiana	121	2.4%	2.0%
Iowa	46	0.9%	0.9%
Kansas	39	0.8%	0.9%
Kentucky	91	1.8%	1.3%
Louisiana	56	1.1%	1.3%
Maine	19	0.4%	0.4%
Maryland	103	2.0%	1.8%
Massachusetts	117	2.3%	2.1%
Michigan	164	3.3%	3.0%
Minnesota	97	1.9%	1.7%
Mississippi	35	0.7%	0.9%
Missouri	67	1.3%	1.8%
Montana	8	0.2%	0.3%

Nebraska	34	0.7%	0.6%
Nevada	54	1.1%	0.9%
New Hampshire	21	0.4%	0.4%
New Jersey	117	2.3%	2.8%
New Mexico	27	0.5%	0.6%
New York	300	6.0%	6.0%
North Carolina	189	3.8%	3.2%
North Dakota	5	0.1%	0.2%
Ohio	219	4.4%	3.5%
Oklahoma	60	1.2%	1.2%
Oregon	103	2.0%	1.3%
Pennsylvania	275	5.5%	3.9%
Rhode Island	18	0.4%	0.3%
South Carolina	69	1.4%	1.6%
South Dakota	11	0.2%	0.3%
Tennessee	105	2.1%	2.1%
Texas	384	7.6%	8.6%
Utah	36	0.7%	0.9%
Vermont	12	0.2%	0.2%
Virginia	148	2.9%	2.6%
Washington	123	2.4%	2.3%
West Virginia	23	0.5%	0.5%
Wisconsin	100	2.0%	1.8%
Wyoming	6	0.1%	0.2%
Puerto Rico	0	0.0%	1.0%

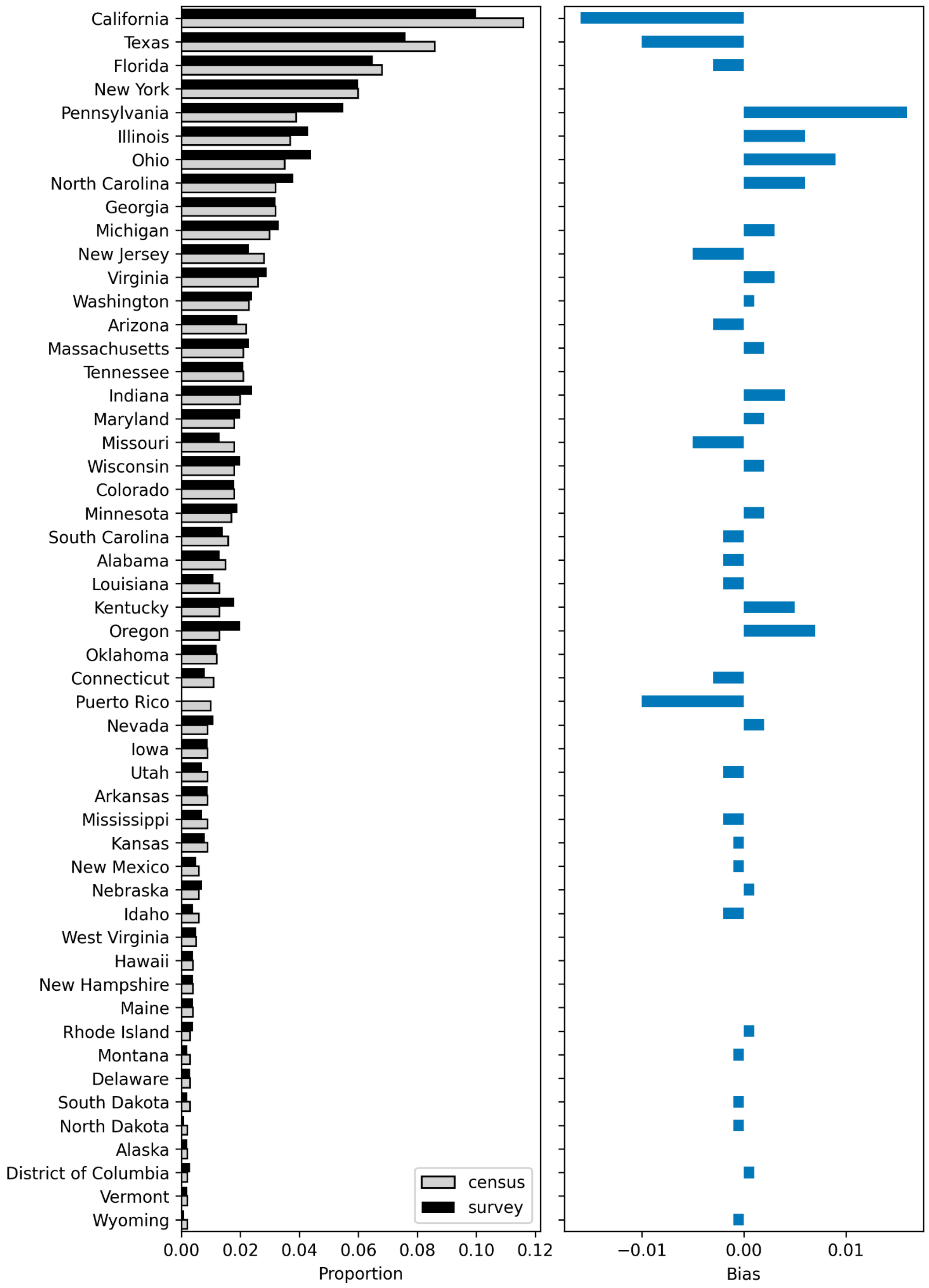

Figure B.1. Sample geographic distribution compared to census population estimates. Left: Proportion of survey sample and US population living in each state. Right: Bias computed as sample proportion minus census proportion.

C. Stratified sampling

Analyses in this work use a randomly stratified sample. Strata were defined for 6 age x sex groups. A binary definition of sex (versus non-binary gender) was used in order to match census data (Male, Female), where users reporting "Other" or "Prefer not to say" in their survey responses were excluded from the stratified sample. Age groups were defined with 3 levels: 18-34, 35-54, 55 and older. This results in 6 strata. The sample was drawn by mapping users to the age x sex stratum matching their survey response and then randomly sampling, without replacement, to create a panel where strata proportions match proportions reported in 2022 U.S. Census data[48]. This results in a stratified sample of size N=1,326. Fig. C.1 displays the sample bias when the stratified sample is not used. It compares the proportion of the 6 strata in the full data sample to the 2022 U.S. Census data.

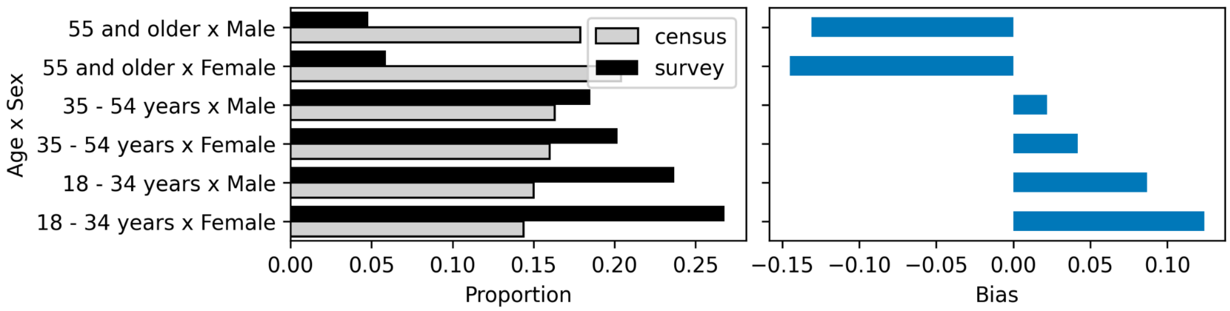

Figure C.1. Sample bias without stratified sampling. Comparison of the 6 strata defined by (Age x Sex) in the full data sample compared to U.S. Census data estimates.

D. Footwear Analysis Robustness Check

This section includes a robustness check for the footwear purchases analysis, where analysis is recomputed by using the stratified sample (N=1,326). The stratified sample is described above (SI C).

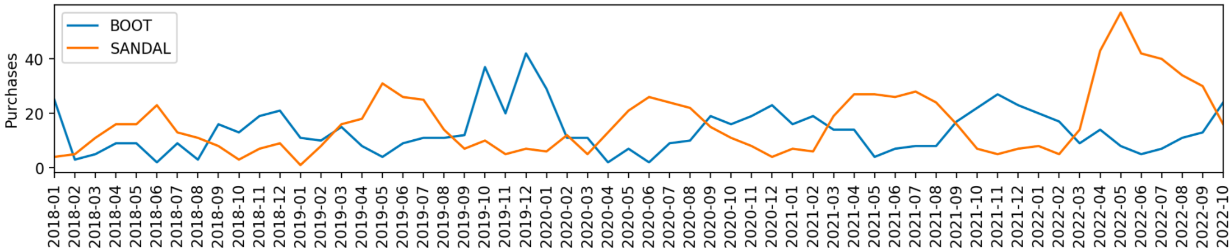

Figure D.1. Total purchases each month for categories BOOT and SANDAL using the stratified sample. Purchases for these products demonstrate different seasonality trends present in the dataset, where SANDAL purchases have yearly peaks in the summer months while BOOT purchases have yearly peaks in the winter months.

E. Books analysis details and robustness check

This section includes details about the book purchases analysis that compares monthly spend on books by users in the Amazon dataset to monthly retail sales from book stores. The retail sales data are collected by the U.S. Census Bureau through their monthly retail trade survey[54]. To compile the set of book purchases from the Amazon data, we include all purchases where the Category column is either "ABIS_BOOK", "BOOK", "BOOKS_1973_AND_LATER". User spend on books is then computed as a monthly summation over the unit price x quantity for each book purchase. This includes 82,954 book purchases from N=4180 distinct users. Fig. E.1 shows a comparison of the monthly spend by all users in the sample versus users in the stratified sample, displaying how these time series are highly similar.

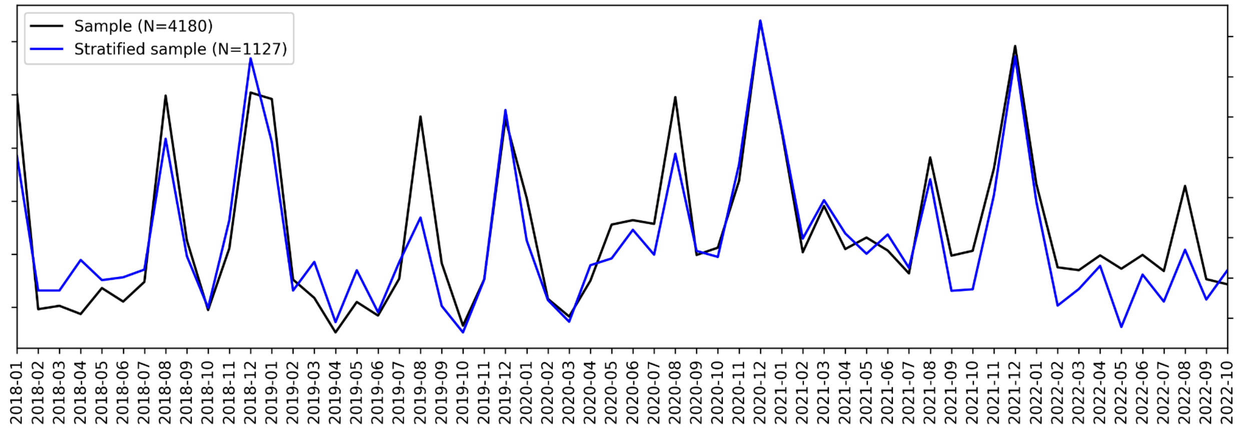

Figure E.1. Monthly spend on books by users in the dataset, comparing spend by all users in the sample to users in the stratified sample.

Fig. E.2 shows the monthly retail sales from book stores compared to monthly spend on books in the Amazon dataset, limited to the stratified sample. This provides an illustrated robustness check and point of comparison to Fig. 6 in the main text.

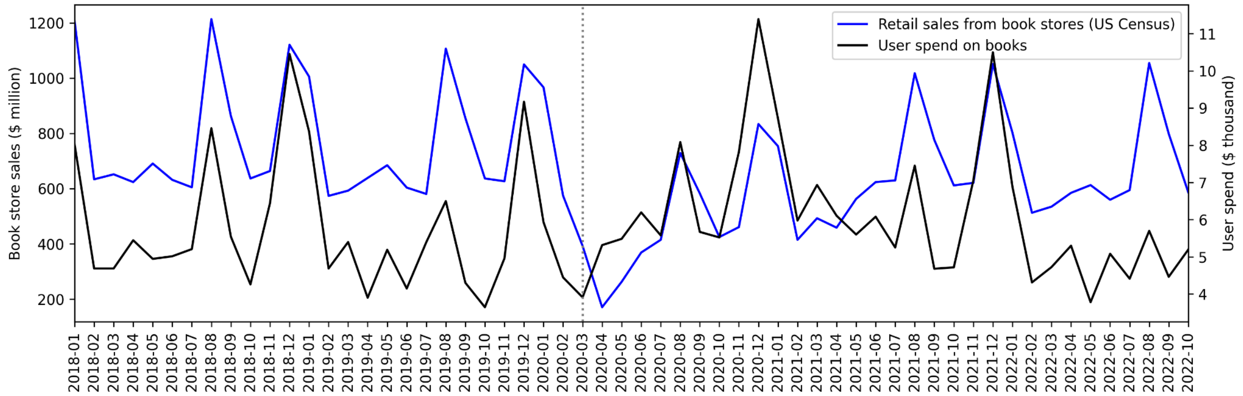

Figure E.2. Monthly retail sales from book stores (collected by the U.S. Census Bureau) compared to monthly spend on books in the Amazon dataset, limited to the stratified sample.

We run an OLS linear regression, as defined in the following equation:

retailSales ~ intercept + postCovid + userSpend

Where

- retailSales is the monthly retail sales data from book stores collected by the U.S. Census Bureau, scaled to millions of dollars.
- userSpend is the monthly total spend on books products by users in our dataset, summed over unit price x quantity, scaled to thousands of dollars.
- postCovid is boolean variable taking the value of 0 if before 2020-03, 1 otherwise.

Results are shown in Table 13 in the main text. Without the stratified sample, R-squared=0.630. With the stratified sample, R-squared=0.555 and all variables remain statistically significant at the $p<0.001$ level.

F. COVID-19 and mask purchases

Our analysis with COVID-19 data and mask purchases compares COVID-19 data to a total number of face mask purchases each month (Fig. 5).

When compiling the face mask purchases data, work was done to filter for these purchases. The intention was not to create a completely exhaustive or precise set of face mask purchases. The intention was to select for enough face mask purchases in order to identify trends in purchases. To compile the face mask purchases, we include all purchases that meet the following criteria:

(a) The product Title contains any of the keywords: "face mask", "facemask", "kf94", "n95" and "mask", and also does not contain any of the keywords "party", "costume", "facial".

(b) The Category matches one of the following categories: "SAFETY_MASK", "RESPIRATOR", "APPAREL_HEAD_NECK_COVERING", "HAT", "PROFESSIONAL_HEALTHCARE", "SPORT_FACE_MASK", "SAFETY_SUPPLY", "FACE_SHIELD", "LAB_SUPPLY".

The analysis could have instead used the number of distinct users making the mask purchases each month. Fig. F.1 shows these metrics have nearly identical trends.

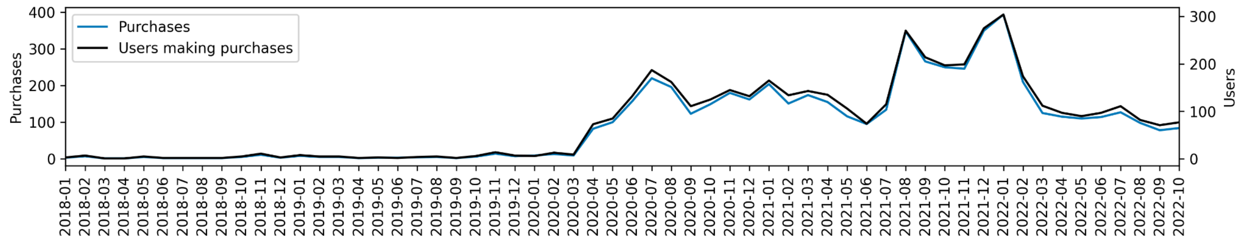

Figure F.1. Monthly total face mask purchases compared to monthly number of distinct users making face mask purchases.

The analysis uses data for reported COVID-19 deaths for the entire U.S., provided by WHO[53] and then aggregated by month. We use data for reported deaths instead of reported cases because COVID-19 testing was limited at the start of the pandemic[64,65], likely resulting in under reported cases. Fig. F.2 shows both the monthly reported COVID-19 cases and deaths data, demonstrating how trends in these metrics are more similar following a period when COVID-19 testing rates increased.

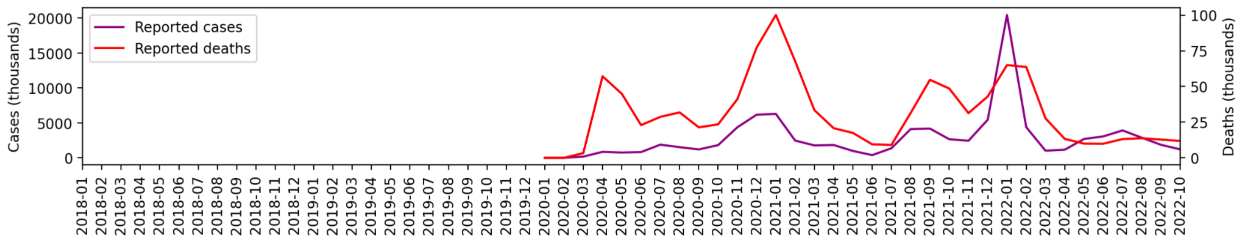

Figure F.2. Monthly reported COVID-19 cases and deaths for the entire U.S.

G. Domestic migration analysis details

We infer each user's U.S. state of residence for each year in order to then infer when users change their state and region of residence between years.

To infer each user's U.S. state of residence in a given year, we first map each month in their purchasing history to the most frequently used shipping address state for that month, and then use the state that is most frequent across those months for the given year.
We exclude participants who do not have purchases with shipping address states for the given year.
We test this methodology against data directly collected by our survey.
Our survey asked participants: "In 2021 which U.S. State did you live in?". When we use the Amazon data to infer state of residence for 2021 and compare inferred state to participants' survey responses for this question. There is a match rate of 96.4% (N=4487 participants with 2021 purchases data).

Each U.S. state is contained within one of 4 regions, as defined by the U.S. Census Bureau[66]. The U.S. Census Bureau collects and publishes information about yearly domestic migration between regions through the American Community Survey (ACS)[55]. This results in 12 data points of population flows between regions for each year. We map users' states of residence inferred from their Amazon purchases to their containing region in order to compare to the census regional domestic migration data. We use regions instead of U.S. states because our dataset is relatively small considering we use a stratified sample and there are 50 states, and a small portion of the population moves each year (<2% of the U.S. population moved between regions in 2018[55]).

When conducting this analysis, we use 2018 to 2019 migration data. We use these years because at the time of analysis, U.S. census data for domestic migration is only available for years up to 2021, and data for 2020, including migration does for 2019 to 2020 and 2020 to 2021 may have been impacted by COVID-19 due to high non-response rates, as noted by the U.S. Census Bureau[67].

We use stratified random sampling, defining strata as elsewhere in this paper: by 3 age groups (18 - 34 years, 35 - 54 years, 55 and older) and 2 groups for sex (male, female), resulting in 6 strata.
We use stratified random sampling to draw a sample matching 2022 U.S. Census data[48]. We resample 1000 times and compute statistics as the mean across the 1000 stratified samples. We compute the portion of users who moved between the 4 regions, for each of the 12 potential flows, between 2018 and 2019. When compared to the 2018 to 2019 census data there is a Spearman correlation coefficient of $r=.830$, $p=.001$.

To further test for sample bias and validate our methodology, we again compare our sample data to census data. In particular, given our data is sampled from a population of crowdworkers, we must ask: Does our sample of crowd worker participants move more often than the general population? Our survey asked participants whether they "Moved place of residence" in 2021. We note this could include any move, and is not restricted to a move across states. We use our stratified sampling process to estimate the percentage of users who moved in 2021 based on response to this question. The result is 16%. The U.S. Census Bureau estimates 12.8% people moved place of residence in 2021[68].

H. Gift card purchases

Fig. H.1 shows the number of gift card (GC) purchases for the top 3 most frequently purchased denominations, while Fig. H.2 shows similar information for the top 8 most frequently purchased denominations. Data users might note spikes in purchases for unusually small denominations, such as $0.50 and $1.00.

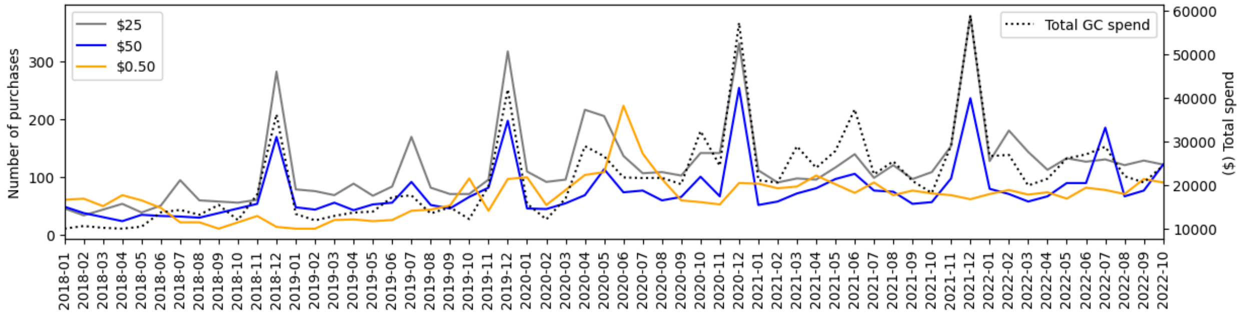

Figure H.1. Monthly gift card (GC) purchases for the top 3 most frequently purchased denominations. The legend is sorted by frequency.

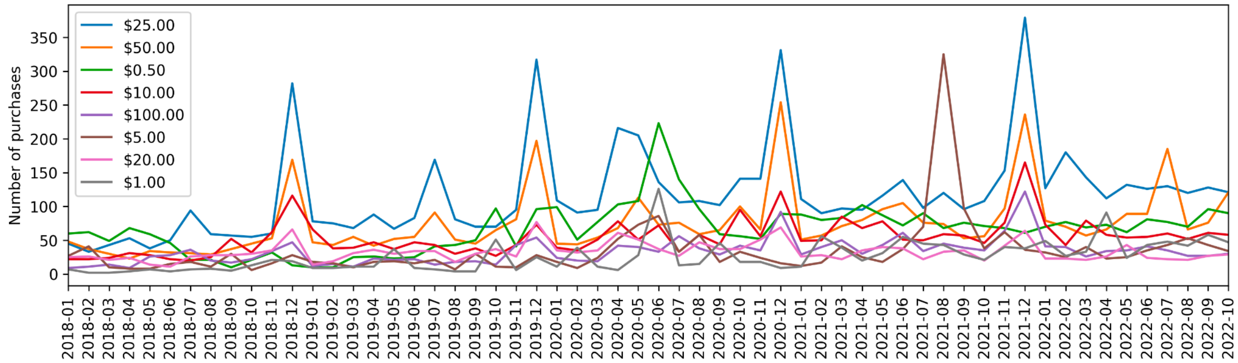

Figure H.2. Monthly gift card (GC) purchases for the top 8 most frequently purchased denominations. The legend is sorted by frequency.

I. Footwear prices compared to footwear CPI

This subsection has details about the time series we used when comparing the monthly median price for footwear purchases in the dataset to the monthly footwear CPI.

For the footwear purchase prices monthly time series: We collected footwear purchases as all purchases with `Category' matching any of: `SHOES', `TECHNICAL_SPORT_SHOE', `BOOT', `SANDAL', `SLIPPER'. We then grouped the purchases by month and computed the median over the unit prices (`Purchase Price Per Unit' column).

For the Footwear CPI time series we use data published by the Bureau of Labor Statistics for the "Consumer Price Index for All Urban Consumers: Footwear in U.S. City Average (CUUR0000SEAE)" that is not seasonally adjusted[63].

These metrics are correlated with a Pearson coefficient of r=0.536, (p<.001).
We also ran an OLS regression, defined by equation (2):

$$footwearCPI \sim intercept + footwearPrices + postCovid + t \quad (2)$$

where:

- footwearCPI takes the monthly CPI value published by the BLS for Footwear, not seasonally adjusted
- footwearPrices is the monthly median unit price computed over all footwear product purchases
- postCovid is boolean capturing changes due to COVID-19, defined as 0 before 2020-03, 1 otherwise
- t is added to capture time based trend, defined as 1,2,3,... for each month in the timeseries data

Results are shown in Table I.1.

Table I.1. OLS regression results for equation (2) predicting the CPI for Footwear (data from U.S. Bureau of Labor Statistics) from median prices for footwear product purchases for N=58 monthly observations.

	Coefficient	Std. error	p
intercept	84.754	38.183	0.000
footwearPrices	0.444	6.678	0.000
postCovid	-5.204	-6.320	0.000
t	0.247	10.222	0.000
R-squared=0.793			

Additional References

64. Mathieu, E. *et al.* Coronavirus Pandemic (COVID-19): Daily new COVID-19 tests per 1,000 people. *Our World Data* (2020).

65. Hasell, J. *et al.* A cross-country database of COVID-19 testing. *Sci. Data* **7**, 345 (2020).

66. U.S. Census Bureau. Census Regions and Divisions of the United States. (2023).

67. U.S. Census Bureau. Changes for 2020 ACS 1-Year Estimates. *Census.gov* https://www.census.gov/programs-surveys/acs/technical-documentation/user-notes/2021-02.html (2021).

68. U.S. Census Bureau. American Community Survey, 1-year estimates, Tables B07001 and DP02.
